# The Response Surface Methodology for Assessment of HLB Values of Mixtures of Non-Ionic Surfactants Using Parameters from Their π-A Isotherms

**DOI:** 10.3390/molecules29102351

**Published:** 2024-05-16

**Authors:** Remigiusz Zapolski, Witold Musiał

**Affiliations:** Department of Physical Chemistry and Biophysics, Wroclaw Medical University, Borowska 211A, 50-556 Wrocław, Poland; remigiusz.zapolski@student.umw.edu.pl

**Keywords:** response surface methodology, hydrophilic-lipophilic balance, sorbitan esters, non-ionic surfactants, π-A isotherm, Langmuir trough, surfactants blend

## Abstract

The aim of the work was to determine important parameters of the course of π-A isotherms, which can determine the HLB (hydrophilic-lipophilic balance) value of surfactant mixtures with selected structural features, such as a straight or branched hydrocarbon chain and a double bond, using RSM (response surface methodology) computational methods. Mixtures of surfactants derived from fatty acids and sorbitan with specific HLB values were evaluated by Langmuir trough. The resulting elasticity modules (ELM) and molecules surfaces (SAM) were evaluated via response surface methodology and respective equations were calculated. The π-A isotherm determined in a Langmuir trough and the ELM and SAM parameters determined on the basis of this isotherm may be useful for determining the HLB of a fixed surfactant mixture. The RSM method used, in which ELM and SAM were assumed as two independent variables, can be a useful technique for tracking the influence of individual molecular characteristics on the hydrophilic-lipophilic properties of mixtures of surfactant compounds. Changes in HLB as a dependent variable can be described as a function of ELM and SAM.

## 1. Introduction

The hydrophilic and lipophilic properties of chemical compounds are determined by the shares of structural components and functional groups that are assigned certain polar or nonpolar properties. As a result, the so-called hydrophilic-lipophilic balance (HLB) number, which is an important parameter that allows determining the hydrophilic-lipophilic properties of various molecules, can be determined. Correct determination of the HLB value is particularly important in the production of ointments, emulsions, and creams administered to the skin. Ointments are systems in which the HLB value of the emulsifier used is particularly important, because the determined HLB value of the surfactant used in the preparation determines the possibility of creating an emulsion and often determines its durability [1]. Complex emulsifiers, which are mixtures of simple emulsifiers, pose particular difficulties when determining the HLB value. Due to potential interactions between molecules, both in the lipophilic and hydrophilic phases, the resulting HLB may differ from the values calculated theoretically based on the mole shares of the components in the surfactant mixture [2]. Moreover, the lipophilic-hydrophilic balance is also influenced by the polar and non-polar phases. Also, in the case of the synthesis of new surfactants, effective methods for determining the HLB value are sought [3]. According to Davis et al., this number can be described by a formula that includes the so-called group numbers related to hydrophilic (γ_H_) and lipophilic (γ_L_) properties [4]. HLB values can be derived by means other than the structural properties of selected functional groups. One such method is the use of magnetic resonance, according to the equation containing the sum of the values of the signals of hydrogens of hydrophilic groups (φ_H_) and the sum of the values of signals of hydrogens of hydrophobic groups (φ_L_) [5]. Another way to determine HLB is the gas chromatography method, which uses the concept of the polarity index (*IP*) defined by the formula taking into account the carbon number (*C*), i.e., the value corresponding to the place where the methanol peak appears in the chromatogram under the influence of some other substance [6]. In the case of some surfactants, HLB is determined based on the ratio of the ethylene oxide content (*E*) to the content of other hydrophilic groups (*P*), or the ratio of the saponification number (*S*) of the tested surfactants to the neutralization number of fatty acids (*A*) [7]. Table 1 presents the most commonly used physicochemical techniques for determining HLB values, along with the equations used to estimate HLB values. Langmuir troughs are used to create, modify, and study monolayers at the gas-liquid or liquid-liquid interface. Langmuir films can be defined as an insoluble monolayer of functional molecules, nanoparticles, that are located at the gas-liquid or liquid-liquid interface. Molecules can move freely at the phase boundary, which makes it possible to control the packing density and study the behavior of the monolayer. The Langmuir isotherm (π-A) allows us to conclude how molecules are packed during the formation of a monolayer. The surface pressure isotherm can also provide a measure of the average surface area per molecule and the compressibility of the monolayer [8]. Langmuir monolayers are obtained by applying a solution of a substance forming a monolayer in a volatile organic solvent to a free surface of water or an aqueous solution. After evaporation of the solvent, an insoluble monolayer is formed on the water surface. The Langmuir trough generates compression of the monolayer using movable barriers. When the monolayer is compressed, the surface pressure changes. The surface pressure is determined using a tensiometer, in which the sensing element is a plate, ring, or wire oriented perpendicular to the water/air interface and partially immersed in the sub-phase. The course of the π-A isotherm depends on what type of chemical compound forms the Langmuir monolayer and on the composition of the subphase. When the monolayer is compressed, the molecules come closer to each other, and the first type of gas-expanded liquid phase transition occurs. With further compression, another phase transition of the first type may occur, i.e., between liquid expanded (LE) and liquid condensed (LC) states.

Due to the widespread use of the Langmuir trough as a research tool, it would be interesting to find a relationship between the π-A isotherm parameters and the HLB value. To the authors’ knowledge, the available literature lacks information on the relationship between the results of Langmuir measurements and the HLB value of the surfactant mixture. Important and measurable parameters obtained in Langmuir measurements of π-A isotherms are the elastic modulus (ELM) and the surface area occupied by the molecule (SAM). Investigating the impact of these parameters on the HLB value could help to understand phenomena occurring at the phase boundary, such as the interaction of lipophilic chains with the same or different conformations. From a practical point of view, this would allow the results from Langmuir isotherm measurements to be used to accurately determine HLB values. This would be directly useful in the design of new emulsifying mixtures intended for use in the preparation of drugs applied to the skin. It should be emphasized that the development of the research method at the current stage is based on a model system of a monolayer of a surfactant mixture and model phases: a hydrophobic air phase and a hydrophilic water subphase. The concept of testing the elasticity module was presented by other authors, including on the example of research on surfactants that are components of the bacterial cell membrane [9]. The π-A isotherm sections, representing the appropriate LC and LE isotherm courses, enable the determination of ELM and SAM. ELM is usually calculated as the ratio of the infinitesimal increase in pressure (∆π) to the resulting relative reduction in area (∆S) [10]. Drug form technology uses numerous surfactants with a defined chemical structure, thereby enabling the creation of drug forms with the desired properties. One of the interesting series of surfactants is a series known in pharmaceutical and cosmetic technology as the Span series. This series can, for example, be ordered according to decreasing HLB value, which is a factor that informs about the hydrophilic and lipophilic properties of the molecule. Sorbitan esters, also known as Spans, are non-ionic surfactants that are used as emulsifying agents in the preparation of emulsions, creams, and ointments for pharmaceutical and cosmetic applications. They form stable water-in-oil emulsions and are often used in various proportions to produce water-in-oil or oil-in-water emulsions or creams with various textures and consistencies. Sorbitan esters are also used as emulsifiers and stabilizers in food. A series of fatty acid esters of sorbitan with decreasing HLB is shown in Table 2.

The representative of the Spans with lowest technical number is Span 20, due to its relatively high HLB, which was not evaluated in the present research. It was applied in niosomes formulations [11,12]. Span 40 was used in the cutaneous form of proniosomes containing tretinoin [13]. Span 60 was used in the formulation of microspheres containing ibuprofen [14]. Span 65 has slightly different applications and is used in formulations used on the skin surface that contain natural products including cinnamon leaf oil [15]. The use of Span 85 is suggested in drug forms containing betamethasone [16] and curcumin [17]. Span 80 mixed with Tween 80 was used in preparations containing fish oil extracts and honey [18]. In the case of mixtures of surfactants, the HLB value may be influenced to varying degrees by both the ELM experimental parameter and the SM experimental parameter. A computational research method that can be effectively used to study the influence of two independent parameters on one dependent parameter is response surface methodology (RSM), which is based on multivariate regression. It enables a statistical assessment of the significance of the impact of the interaction of individual variables on the dependent parameter. In the field of pharmaceutical technology, RSM has been used in the optimization of ultrasound-assisted enzymatic extraction of active ingredients from natural raw materials and the analysis of the release of substances from the drug matrix [19]. HLB value is a variable depending on the structure of the molecule and its specific properties, including the presence of double bonds and carbon chain branches. The structural variables of the tested molecules are characteristic for the parts exposed above the subphase during the monolayer building process, as well as the parts immersed in the subphase. Part of a molecule that is hydrophilic anchors the molecule in the subphase. Based on the studies of Langmuir monolayers [8], an interaction between lipophilic and hydrophilic structures within a given compartment may be assumed. The assumption of interactions resulting from specific features of the molecular structure and expressed in their mechanical properties may be analogous to surfactant-co-surfactant interactions in dispersion systems [20].

The aim of the work was to apply important parameters of the course of π-A isotherms to determine the HLB values of surfactant mixtures with selected structural features, such as a straight or branched hydrocarbon chain and a double bond, using RSM computational methods.

The selection of the homologous series of tested surfactants provided the invariance of the hydrophilic part located in the subphase, what enabled evaluation of interactions between molecular components located above the subphase; the molecules parts immersed in the subphase were merely hydrophilic sorbitan groups. It has been proposed to study surfactant systems with different carbon chain lengths, with or without double bonds within the carbon chain, and having one or three carbon chains within the tested molecule.

The statistical analysis included two variables that could influence the HLB value of the part above the subphase: the elasticity coefficient (ELM) of the monolayer and the surface area per molecule (SAM).

## 2. Results

### 2.1. Experimentally Determined ELM and SAM of the Tested Surfactant Mixtures

Table 3 contains the determined elasticity coefficients and the estimated surface area per molecule for the LC and LE states assessed using a Langmuir trough in a separate series of experiments. The presented values were obtained from π-A isotherms for pure surfactants and their mixtures. The S40/S80 mixture had the highest value of surface area per particle in the LE state, while the S60 preparation had the lowest value. Pure S40 had the highest absolute value of the ELM in the LE state, while the S40/S83 system had the lowest absolute value. In the case of the LC state, the largest surface area per molecule was observed in the S40/S83 system, whereas the lowest value was observed in the S40/S65 system. In the case of the modulus of elasticity in the LC state, the pure S60 system had the highest absolute value, while the S40/S83 system had the lowest absolute value.

### 2.2. HLB as a Function of ELM and SAM in RSM Model

Table 4 presents multivariate regression coefficients for the independent variables SAM and ELM for the equation presented in general form, with HLB as dependent variable, according to Section 4.4.1.

The highest value of the intercept β_0_ was recorded for the LC state in the case of Equation (4) (38.711) and for the LE state in the case of Equation (5) (64.979). The negative value of the intercept for the case of Equation (3) (−54.336) corresponding to the LE phase should be noted. The coefficient reached its highest value in the case of Equation (4) (126,731.830) for the LC state and in the case of Equation (5) (134,200.582) for the LE state, while the lowest values corresponded to the cases of Equation (2) (−7490.891) for the LC phase and Equation (1) (−704.538) for the LE state. At the same time, it is worth emphasizing the negative value of the coefficient for the cases of Equation (1) (−43.791) and Equation (2) (−722.783) for the LC state and Equation (1) (−704.538) and Equation (3) (−101954.040) for the LE state. In the case of the SAM coefficient, the highest value was recorded for Equation (2) (0.214), while the smallest in the case of Equation (3) (−0.4909) for the LC state and the smallest in the case of the LE state for Equation (2) (0.071), whereas the highest for the Equation (5) (1,308,742) case. For the LC state ELM-ELM coefficient, the highest value was recorded in the Equation (4) (126,731.830), the smallest for the case of Equation (2) (−722.783), while in the case of the LE state the highest value was characteristic of case Equation (2) (134,200.582) and the smallest Equation (3) (−101,954.040). The SAM-SAM parameter for the case of Equation (5) during the calculations was not determined for both the LE and LC states, while the highest value for the LC state was achieved in the case of Equation (4) (0.00324) and the smallest in the case of Equation (1) (0.00014); for the LE phase the highest value is characteristic of the case Equation (3) (0.00996) and the smallest Equation (2) (0.0002).

**Figure 1:**
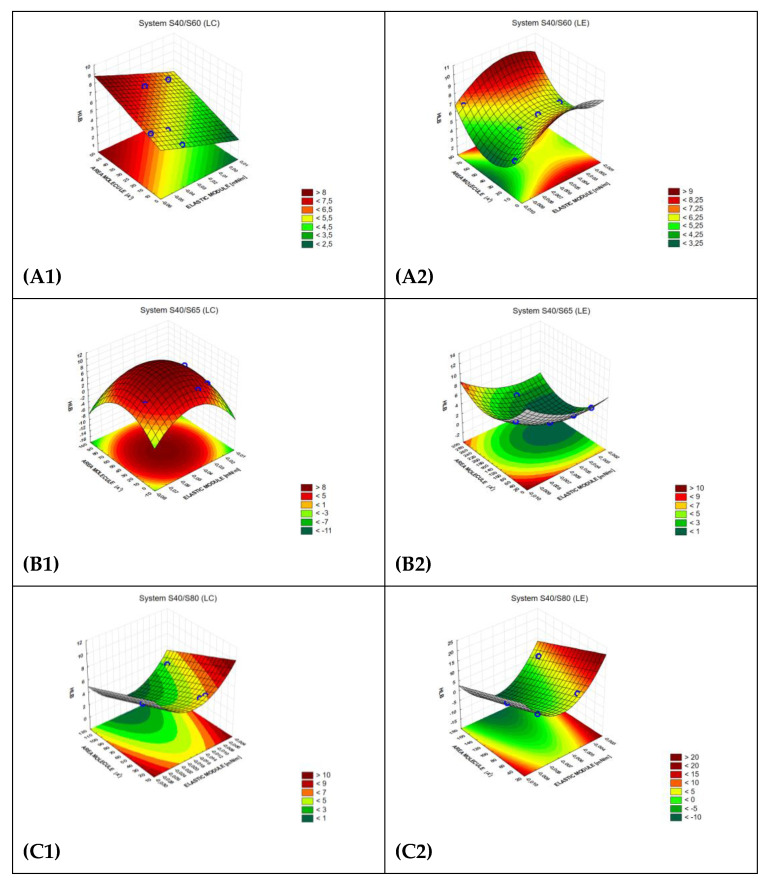

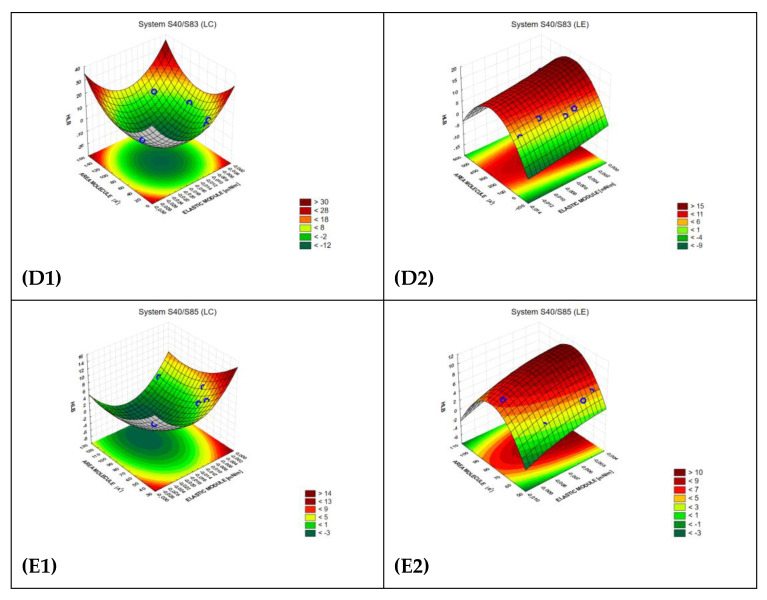
The visualization of the influence of two experimentally assessed independent parameters—predictors, i.e., area of the molecule (SAM in the text) and elastic module (ELM in the text), on the level of dependent parameter: HLB value, part 1: systems S40/S60 (**A1,2**), S40/S65 (**B1,2**), S40/S80 (**C1,2**) S40/S83 (**D1,2**), S40/S85 (**E1,2**) in liquid condensed state (LC) and liquid extended state (LE)—1 and 2 no respectively.

The models calculated above were visualized on the following Figure 1. In cases B1, D2, and E2, the surface response was convex, while for cases C1, C2, A2, B2, D2, and E2, the surface response was concave.

### 2.3. The Statistical Significance of the Influence of the Independent Variables ELM and SAM on the Dependent Variable HLB

The T statistics values for assessed systems are presented in Table 5. Asterix (*) denominates statistically significant values of T-score of statistics regression parameters for evaluated bi-surfactant systems.

In the case of the S40/S60 preparation, the parameters of the SAM equation in a linear relationship for the LE state (T_LE_ = 1.98) and ELM in a linear relationship for the LC state (T_LC_ = 2.42) were characterized by statistical significance. In the case of the S40/S65 mixture, the statistically significant parameters of the equations were the ELM parameter for the LE state (T_LE_=4.27) in a linear relationship and the ELM parameter in a quadratic relationship for the LE (T_LE_ = 9.59) and LC (T_LC_ = 3.38) states. For the S40/S80 mixture, the SAM parameter was statistically significant for the LC (T_LC_ = 3.79) and LE (T_LE_ = 10.9) states in a linear relationship, and also the ELM parameter in the quadratic relationship for both states (T_LC_ = 5.45; T_LE_ = 22.01). For the S40/S83 mixture, the statistically important parameters were SAM (T_LC_ = 9.13; T_LE_ = 3.00) and ELM (T_LC_ = 3.92; T_LE_ = 2.52) in a linear relationship for both phases and ELM in a quadratic relationship for the LC state (TLC = 6.31). For the research system of the S40/S85 mixture, the parameter SAM in the linear relationship were statistically significant for both states (T_LC_ = 2.25; T_LE_ = 5.43) and ELM in the case of the LE state (T_LE_ = 2.07). The SAM parameter in the quadratic relationship for the LE state was on the border of statistical significance (T_LE_ = 1.97).

## 3. Discussion

### 3.1. General Remarks on Structure of the Surfactants an Applied Equation

Molecules containing shorter carbon chains tend to be partially immersed in the subphase, as was presented in available bibliography [21,22]. Based on the structures of the tested compounds, a model distribution of particles of surfactant mixtures in the monolayer structure at the interfacial area was proposed and is discussed below. The patterns of the proposed layout of the surfactants particles were presented in short in Table 6.

### 3.2. The Influence of Regression Parameters on HLB Value and the Structural Layout of Surfactants in Interfacial Area

The multivariate regression equation (Equation (6)) in generic form contained the free term ($\beta_{0}$) as well as regression parameters ($\beta_{ELM}$, $\beta_{SAM}$, $\beta_{ELMELM}$, $\beta_{SAMSAM}$), which were analyzed in detail.

(6) $$HLB=\beta_{0}+\beta_{ELM}x_{ELM}+\beta_{SAM}x_{SAM}+\beta_{ELMELM}x_{ELMELM}^{2}+\beta_{SAMSAM}x_{SAMSAM}^{2}+\beta_{ELMSAM}x_{ELM}x_{SAM}+\varepsilon$$

Free term (*β*_0_)

Equations (1), (2), and (5) are characterized by values of the intercept (*β*_0_) in the range from 2.382 to 13.627 for the LC phase and in the range from 7.426 to 8.226 for the LE phase, except for the equation Equation (5). These values contrast with the values of the intercept term in Equation (3) and Equation (4) for the LC phase: 22.44 and 38.711, respectively. In the case of Equation (3), in the LC, close to the HLB value of the tested systems, the equation indicates an insignificant impact of the tested parameters *β_ELM_, β_ELM ELM_, β_SAM SAM_* on the estimated resulting HLB value of the mixture (Table 4). It should be noted that a high value of the free term characterizes the mixture systems in which the added surfactant is characterized by specific structural properties, such as the presence of a double bond or the presence of more than one carbon chain determining hydrophobic properties.

SAM coefficient ($\beta_{SAM}$, $\beta_{SAMSAM}$)

The value of the $\beta_{SAM}$ coefficient in all equations oscillated in the range of −0.4909–0.214 for the LC phase and 0.071–1.601 for the LE phase, except Equation (5), in which $\beta_{SAM}$ was strongly deviated from the others and amounted to 130,8742. Except for this equation (Equation (5)), the absolute values of $\beta_{SAM}$ were less than 1, which allows us to assume that $\beta_{SAM}$ only slightly determined the value of the resulting HLB in the case of both the LE and LC phases. Note that the high value of the $\beta_{SAM}$ coefficient in Equation (5) corresponds to the inability to determine the $\beta_{SAM\;SAM}$ ratio for both the LC and LE phases, and a characteristic feature of the doped S80 surfactant is a double bond in a single carbon chain. In this system (S40/S80), a high value of the $\beta_{SAM}$ coefficient characterizes the LE phase in which interactions between the subphase and structures oriented towards the air may dominate (Table 6, IA.2). This may be due to the low density of amphiphiles at the phase boundary, compared to the LC phase, which should be dominated by interactions between the hydrophobic components of the molecules. This fact can be attributed to the interactions between the water dipoles from the subphase and the π−electrons of the double bond of the surfactant molecule. In the case of the $\beta_{SAM\;SAM}$ parameter for the LC phase, it ranged from 0.00014 to 0.0027; in the case of the LE phase, it ranged from 0.000139 to 0.0002. A low value of the $\beta_{SAM\;SAM}$ parameter corresponded to a low value of the $\beta_{SAM}$ parameter (Table 4).

ELM coefficient (β_ELM_, β_SAM_, β_ELM ELM_)

The $\beta_{ELM}$ coefficient can be considered a parameter that characterize the mechanical properties of the monolayer during its construction under the influence of the force exerted by the Langmuir balance barriers (Table 4). High values of the $\beta_{ELM}$ parameter correspond to high values of the $\beta_{ELM\;ELM}$ parameter for both the LC and LE phases. Noteworthy is the negative value of this parameter in the Eq equation, as for the LC and LE phases and in Equation (2) case of LC phase. It is also worth noting that negative values of the $\beta_{ELM}$ coefficient accompany equations describing mixtures containing surfactants with straight-chain hydrophobic components. A high value of the $\beta_{ELM}$ parameter may indicate steric repulsion during the construction of the monolayer. A positive high value of the $\beta_{ELM}$ coefficient was recorded in the case of S40/S85 and S40/S83 mixtures, amounting to 1257.341 and 4087.569, respectively. The S40/S85 and S40/S83 systems were characterized by the presence of at least two hydrocarbon chains and a double bond (Table 6, IA.4 and IA.5). In the case of S80, characterized by the presence of a double bond in the only carbon chain ($\beta_{ELM}$ = 882.334 for the LC phase), the high value of the $\beta_{ELM}$ coefficient for the LE phase is noteworthy. Simultaneously, the $\beta_{ELM\;ELM}$ value was low; this may correspond to the high molecular interaction surfactant with the subphase and, at the same time, prove that the $\beta_{ELM\;ELM}\;$ coefficient describes the intermolecular interactions in the created monolayer.

### 3.3. Influence of Equation Parameters on the HLB Value in the Terms of the T Statistics

Presented regression equations (Equation (1), Table 4) had an interchangeable linear influence on the predicted HLB value. The appropriate values of the “T” statistics parameter for the research system S40/S60 mixture (Table 5; *T_LC_* = 2.42) of $\beta_{ELM}$ Table 4, ($\beta_{ELM}=-43.791)$ were determined in the LC state with a linear negative relationship. The $\beta_{SAM}$ ($\beta_{SAM}=0.144)$ had a positive impact on HLB in the LE state (Table 5; T_LE_ = 1.98). This effect can be attributed to the fact that the hydrophobic chains in both surfactants are simple saturated hydrocarbon chains (Table 6, IA.1).

Similar to the case of the S40/S60 mixture, there was also a significant positive influence of the ELM (Table 5, T_LC_ = 4.7) parameter, tested in the LE (Table 4, $\beta_{ELM}=968.807)$ state in the S40/S65 mixture, but in a quadratic relationship, according to the equation Equation (2) (Table 4, $\beta_{ELM\;ELM}=134200.582$). In the LE state, unlike in S40/S60, the ELM component was important, both in the linear and quadratic relations (T_LE_ = 9.59, T_LC_ = 3.38). It seems that there was a significant effect with a positive trend of ELM ($\beta_{ELM\;}=968.807$) on HLB value in the LE state but with a negative trend in the LC state ($\beta_{ELM\;}=-722.783$). The branched structure of the carbon chain was responsible for changing the mechanical properties (elastic modulus) of monolayer (Table 6, IA.3–IA.5).

The use of a component containing a double bond in the chain, i.e., S80, in the surfactant mixture was associated with the reveal of a significant impact on the estimated HLB value of both parameters, i.e., ELM (T_LC_ = 22.01, T_LE_ = 5.45) with a linear ${(\beta }_{ELM\;}=882.331)$ and a quadratic $\left(\beta_{ELM\;ELM}=25445.147\right)$ relationship (both positive impact) and SAM (T_LC_ = 3.79, T_LE_ = 10.9) with linear positive impact. It should be clearly noted that the impact of SAM on HLB was significant in a linear relationship, while the impact of ELM remained significant in a quadratic relationship. A branched chain of surfactant structure with a double bond created a mixture in which a significant impact of SAM (T_LC_ = 2.25, T_LE_ = 5.43) with linear negative correlation in LC state and positive linear correlation in LE state on HLB was observed. In only one linear relationship, in the LE state, did ELM (T_LE_ = 4.27) have a significant impact on HLB with negative trend.

An interesting system for research was the S40/S83 mixture, in which S40 was doped with S83, which contains unesterified and esterified oleic acid in a ratio of 1:3. In this particular case, the influence of both SAM (T_LC_ = 3) and ELM (T_LC_ = 2.52) in the LC state with positive linear relationship and ELM (T_LE_ =6.31) in LE state was significant with quadratic positive relationship.

The research system with a large carbon chain component is S40/S85. The predicted value of HLB of this system depended mainly on ELM (T_LC_ = 2.25) in LC state with linear correlation with positive impact ${(\beta }_{ELM\;}=1257.34)$ and SAM (T_LE_ = 5.43) in LE state with linear correlation with negative impact ${(\beta }_{SAM\;}=-0.310)$ and for LE state depended on ELM (T_LE_ = 2.07) in linear negative relationship ${(\beta }_{ELM\;}=-625.619)$ and quadratic relationship but in border of significance (T_LE_ = 1.97).

## 4. Materials and Methods

### 4.1. Materials

Span 40 (Sorbitan Monopalmitate, HLB 6.7), Span 60 (Sorbitan Monostearate, HLB 4.7) Span 65 (Sorbitan Tristearate, HLB 2.1), Span 80 (Sorbitan Oleate, HLB 4.3), Span 83 (Sesqui- Sorbitan Oleate, HLB 3.7), Span 85 (Sorbitan Trioleate, HLB 1.8). All forms of surfactants were purchased from Merck, Warszawa, Poland, with a purity above 99%; deionized water with conductivity below 2 µS/cm was applied. For preparing solutions of surfactant mixtures, chloroform of p.a. purity was used. The Langmuir troughs were cleaned by isopropyl alcohol of p.a. purity.

### 4.2. Preparation of Surfactant Mixtures of Theoretically Determined HLB

In the first stage of the research, the theoretical maximum HLB of the tested surfactant mixtures was calculated as HLB_mx_ (Equation (7)). HLB was treated as an additive value and was calculated based on the mole fraction of the tested surfactants in the mixture, i.e., the main surfactant (f_S40_) and its HLB, to which a specific amount of a second surfactant was added (f_SX_) with a specific HLB (HLB_SX_), according to the formula below and data from Table 7.

HLB_mx_ = HLB_S40_ × f_S40_ + HLB_SX_ × f_SX_ (7)

### 4.3. Experimental Determination of Elasticity Modules and Surface Area per Molecule Values

The elasticity modules (ELM) and the surface area per molecule (SAM) for the condensed liquid state (LC) and for the expanded liquid state (LE) were experimentally determined on the basis of π-A isotherms. The tests used a Kibron MicroTrough (Kibron, Helsinki, Finland), measuring element in the form of a platinum rod, flame cleaned with a gas burner. The experiment was controlled, and results recorded using FilmwareX 4.0. The initial calibration of the device was carried out based on the tabulated value of the subphase surface tension and the standardized detector mass. Deionized water with a conductivity of 5 µS/cm was used as a subphase. The evaluated surfactants were dissolved in chloroform (HPLC grade). The concentration of stock solutions was in the range of 1 mg/mL. The volume of surfactant solutions used was set at 5 microliters. Mixtures with the required molar proportions were obtained by mixing the calculated volumes of stock solutions immediately before applying them to the subphase. All experiments were carried out at an ambient temperature of 25 +/− 1. The volume of the chloroform solution of the tested surfactants and their mixtures was determined at a distance of 5 cm from one of the barriers. The monolayer was conditioned until complete evaporation of the solvent within 15 min. The surface of the subphase was protected against solid particles usually present in the air by an externally applied transparent coating. The barrier speed was set to 14.5 mm/min from the starting position. The segments representing the respective LC and LE courses of isotherms were selected according to the consequent linear parts of the plots for every evaluated monolayer. The sets of points were limited to the areas with high Pearson’s coefficients. The surface areas of individual surfactants particles and the theoretical values for mixtures, in LC and LE states, respectively, were calculated by extrapolating the linear regions of LC and LE isotherms to the x axis at 0 point. The ELM was calculated as the ratio of the infinitesimal pressure increase (Δπ) to the resulting relative decrease of the volume (ΔS), according to Equation (8).

(8) $$ELM=-S\frac{∆\pi }{∆S}$$

### 4.4. Assessment of the Impact of ELM and SAM on the Theoretically Calculated HLB Value Using RSM

#### 4.4.1. Multivariate Analysis

To examine the influence of individual parameters, i.e., ELM and SAM, on the HLB value, the RSM was used, in accordance with the general form of the multivariate analysis formula (Equation (6)).

The above-mentioned parameters, ELM, SAM, and HLB, were introduced into the “Statistica” program—DOE module, central composition plans, as independent variables. The ELM and SAM values were provided from measurements performed according to Section 4.3. The program provided visualizations of the impact of ELM and SAM on the theoretical HLB value of the tested surfactant mixtures. Moreover, optimized surface equations representing the impact of ELM and SAM on HLB were determined. The impact of individual parameters, i.e., ELM and SAM, was examined in detail based on the above-mentioned method. Multivariate regression equations in which HLB is a function of ELM and SAM described by Equation (6) in generic form adopted from general multivariate equation.

#### 4.4.2. T Statistics Values of Evaluated Systems

Using the above-mentioned software package, the values of the “T” statistics were calculated and presented in a Pareto chart. The T values informed about the significance of specific predictors, here ELM or SAM—the independent variables, in predicting the value of HLB—the dependent variable. If absolute T value for the proposed predictor exceeded 2, the examined predictor was accepted as statistically significant factor for prediction of the value of the HLB parameter. The T statistics for predictors ELM and SAM were calculated for the LC and LE states of monolayer separately.

## 5. Conclusions

The π-A isotherm determined in a Langmuir trough and the ELM and SAM parameters determined on the basis of this isotherm may be useful for determining the HLB of a fixed surfactant mixture. The RSM method, in which ELM and SAM were assumed as two independent variables, can be a useful technique for tracking the influence of individual molecular characteristics on the hydrophilic-lipophilic properties of mixtures of surfactant compounds. Changes in HLB as a dependent variable can be described as a function of ELM and SAM. The SEM and ELM values may support the consideration of the structural layout of surfactants particles in monolayers on the interfacial area, i.e., the possible interactions between the various particles. In future research, the authors plan to apply the estimated parameters of the surfactants mixtures for the formulation processes of preparations containing active substances with antifungal or antibiotic properties. The planned research will consider the stability aspects of the formulations in the context of aging phenomena of dispersion systems and hydrophilic-lipophilic equilibria.

## Figures and Tables

**Table 1:** Lists of sample equations reflecting the HLB calculation method.

Parameters Source	Equation
Group numbers related to hydrophilic and lipophilic properties	$HLB=7+\sum_{1}^{i}\gamma_{H}+\sum_{1}^{i}\gamma_{L}$ (1)
Nuclear Magnetic Resonance	$HLB=\frac{15\varphi_{H}}{5\left(15\varphi_{H}+10\varphi_{L}\right)}\;$ (2)
Gas Chromatography	$IP=100\cdot \log\left(C-4.7\right)+60\;$ (3)
Composition	$HLB=\frac{E+P}{5}$ (4)
Saponification number	$HLB=20\left(1-\frac{S}{A}\right)$ (5)

**Table 2:** A series of fatty acid and sorbitan esters with decreasing HLB, along with acronyms used in this research work and simplified chemical names enabling quick identification of the surfactant structure.

No	Trade Name	Simplified Chemical Name	HLB	Acronym
1.	Span 40	Sorbitan monopalmitate	6.7	S40
2.	Span 60	Sorbitan monostearate	4.7	S60
3.	Span 65	Sorbitan tristearate	2.1	S65
4.	Span 80	Sorbitan monooleate	4.3	S80
5.	Span 85	Sorbitan trioleate	1.8	S85

**Table 3:** ELM and SAM of the tested surfactant mixtures calculated from π-A isotherms.

State	LC		LE	
Parameter	ELM	SAM	ELM	SAM
Preparation	[mN/m]	[Å^2^]	[mN/m]	[Å^2^]
S40	−2.73 × 10^−2^	41.92	−9.40 × 10^−3^	76.59
S40/S60	−5.66 × 10^−2^	14.32	−4.90 × 10^−3^	35.82
S40/S60	−1.60 × 10^−3^	45.11	−1.80 × 10^−3^	41.83
S40/S60	−4.09 × 10^−2^	15.21	−7.00 × 10^−3^	34.27
S60	−3.95 × 10^−2^	6.68	−9.60 × 10^−3^	12.20
S40	−2.73 × 10^−2^	41.92	−9.40 × 10^−3^	76.59
S40/S65	−3.72 × 10^−2^	2.77	−5.10 × 10^−3^	18.19
S40/S65	−7.22 × 10^−2^	20.04	−6.70 × 10^−3^	48.76
S40/S65	−2.16 × 10^−2^	16.06	−2.30 × 10^−3^	57.16
S65	−3.72 × 10^−2^	88.76	−5.10 × 10^−3^	272.08
S40	−2.73 × 10^−2^	41.92	−9.40 × 10^−3^	76.59
S40/S80	−1.30 × 10^−2^	22.81	−8.50 × 10^−3^	30.92
S40/S80	−1.19 × 10^−2^	19.13	−5.00 × 10^−3^	32.13
S80	−6.60 × 10^−3^	107.18	−3.60 × 10^−3^	161.19
S40	−2.73 × 10^−2^	41.92	−9.40 × 10^−3^	76.59
S40/S83	−7.40 × 10^−3^	21.87	−4.00 × 10^−3^	28.03
S40/S83	−9.40 × 10^−3^	138.67	−1.10 × 10^−3^	549.39
S40/S83	−9.10 × 10^−3^	16.30	−5.70 × 10^−3^	21.09
S83	−4.80 × 10^−3^	84.21	−1.29 × 10^−2^	47.60
S40	−2.73 × 10^−2^	41.92	−9.40 × 10^−3^	76.59
S40/S85	−2.70 × 10^−3^	67.06	−4.60 × 10^−3^	56.92
S40/S85	−6.00 × 10^−3^	50.39	−5.30 × 10^−3^	55.84
S40/S85	−6.40 × 10^−3^	59.68	−8.10 × 10^−3^	56.69
S85	−4.90 × 10^−3^	125.64	−7.50 × 10^−3^	107.05

ELM—elasticity modulus, LC—liquid condensed, SAM—molecule area, LE—liquid expanded, preparations acronyms described in Table 7 (Section 4.1).

**Table 4:** Multivariate regression coefficients of the adopted model of the impact of ELM and SAM on HLB.

No.	Preparation	LC State					LE State				
		$\beta_{0}$	$\beta_{ELM}$	$\beta_{SAM}$	$\beta_{ELM\;ELM}$	$\beta_{SAM\;SAM}$	$\beta_{0}$	$\beta_{ELM}$	$\beta_{SAM}$	$\beta_{ELM\;ELM}$	$\beta_{SAM\;SAM}$
Equation (1)	S40/S60	2.382	−43.791	0.080	39.031	0.00014	7.426	−704.538	0.144	86,932.866	0.0019
Equation (2)	S40/S65	11.543	−722.783	0.214	−7490.891	0.0027	8.226	968.807	0.071	134,200.582	0.0002
Equation (3)	S40/S85	22.440	1257.341	−0.310	38,875.915	0.00145	−54.336	−625.619	1.601	−101,954.040	0.00996
Equation (4)	S40/S83	38.711	4087.569	−0.4909	126,731.830	0.00324	6.484	619.960	0.0764	11,349.862	0.000139
Equation (5)	S40/S80	13.627	882.331	0.0430	25,445.147	-	64.979	17,820.387	1,308,742	0.0836	-

ELM—elasticity modulus, LC—liquid condensed, SAM—molecule area, LE—liquid expanded, preparations acronyms described in Table 1 shaded values —statistically significant parameters (Pareto charts).

**Table 5:** Presentation of T-score of statistics regression parameters for liquid condensed (T_LC_) and liquid expanded (T_LE_) states of monolayers. ELM—elasticity modulus, SAM—molecule area. Preparations acronyms are described in Table 1.

Preparation	Partial Function	Parameter	State of the Monolayer	
			T_LC_	T_LE_
S40/S60	Linear	SAM	0.02	1.98 (*)
		ELM	2.42 *	0.63
	Square	SAM	0.05	0.05
		ELM	0.88	0.003
S40/S65	Linear	SAM	1.37	1.28
		ELM	1.02	4.27 *
	Square	SAM	0.92	0.76
		ELM	9.59 *	3.38 *
S40/S80	Linear	SAM	3.79 *	10.90 *
		ELM	0.40	4.68 *
	Square	SAM	N/A	N/A
		ELM	5.45 *	22.01 *
S40/S83	Linear	SAM	9.13 *	3.00 *
		ELM	3.92 *	2.52 *
	Square	SAM	0.25	0.16
		ELM	6.31 *	0.16
S40/S85	Linear	SAM	2.25 *	5.43 *
		ELM	1.22	2.07 *
	Square	SAM	0.21	1.97 (*)
		ELM	0.04	0.40

* statistically significant.

**Table 6:** Proposed model of behavior of mixture components in interfacial area.

Structure of Span Surfactants	Type of Interfacial Area	Proposed Behavior of Mixture Components in Interfacial Area
S40 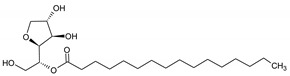	-	Common ingredient for all research systems
S60 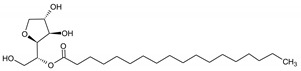	IA.1	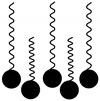
S80 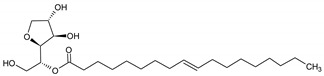	IA.2	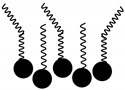
S65 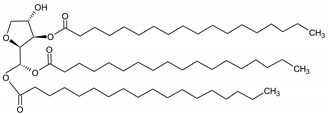	IA.3	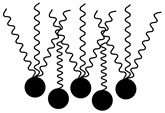
S83 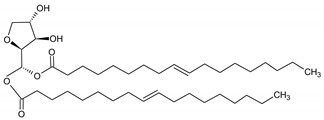	IA.4	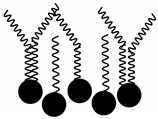
S85 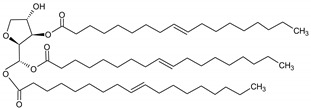	IA.5	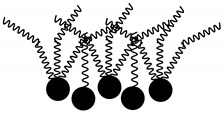

**Table 7:** Composition of the tested surfactant mixtures and calculated HLB values of these mixtures.

S40/S60		HLB_mx_	S40/S65		HLB_mx_	S40/80		HLB_mx_	S40/S83		HLB_mx_	S40/S85		HLB_mx_
f_S40_	f_S60_		f_S40_	f_S65_		f_S40_	f_S80_		f_S40_	f_S83_		f_S40_	f_S85_	
1.00	0.00	6.70	1.00	0.00	6.70	1.00	0.00	6.70	1.00	0.00	6.70	1.00	0.00	6.70
0.70	0.30	6.10	0.75	0.25	5.55	0.49	0.51	5.48	0.84	0.16	6.22	0.70	0.30	5.23
0.53	0.47	5.76	0.58	0.42	4.77	0.67	0.33	5.91	0.72	0.28	5.86	0.54	0.46	4.45
0.36	0.64	5.42	0.26	0.74	3.30	0.00	1.00	4.30	0.39	0.61	4.87	0.20	0.80	2.78
0.00	1.00	4.70	0.00	1.00	2.10	-	-	-	0.00	1.00	3.70	0.00	1.00	1.80

f_S_ represents the molar fraction of respective surfactants in mixtures, as described in the main text, whereas HLB_mx_ represents the resulting value of HLB mixture.

## Data Availability

The data presented in this study are available on request from the corresponding author. The data are not publicly available due to high number of repeated values of consecutive measurements.
